# Editorial: Regulation of Endurance Performance: New Frontiers

**DOI:** 10.3389/fphys.2017.00727

**Published:** 2017-09-21

**Authors:** Florentina J. Hettinga, Andrew Renfree, Benjamin Pageaux, Hollie S. Jones, Jo Corbett, Dominic Micklewright, Alexis R. Mauger

**Affiliations:** ^1^School of Sport, Rehabilitation and Exercise Science, University of Essex Colchester, United Kingdom; ^2^Institute of Sport and Exercise Science, University of Worcester Worcester, United Kingdom; ^3^CAPS UMR1093, Institut National de la Santé et de la Recherche Médicale (INSERM), Université de Bourgogne-Franche Comté Dijon, France; ^4^School of Psychology, University of Central Lancashire Preston, United Kingdom; ^5^Department of Sport and Exercise Science, University of Portsmouth Portsmouth, United Kingdom; ^6^School of Sport and Exercise Sciences, University of Kent Canterbury, United Kingdom

**Keywords:** pacing, endurance, sport performance, training, fatigue, brain, recovery

## Introduction

Successful endurance performance requires the integration of multiple physiological and psychological systems, working together to regulate exercise intensity in a way that will reduce time taken or increase work done. The systems that ultimately limit performance of the task are hotly contested, and may depend on a variety of factors including the type of task, the environment, external influences, training status of the individual and a host of psychological constructs. These factors can be studied in isolation, or inclusively as a whole-body or integrative system. A reductionist approach has traditionally been favored, leading to a greater understanding and emphasis on muscle and cardiovascular physiology, but the role of the brain and how this integrates multiple systems is gaining momentum. However, these differing approaches may have led to false dichotomy, and now with better understanding of both fields, there is a need to bring these perspectives together.

The divergent viewpoints of the limitations to human performance may have partly arisen because of the different exercise models studied. These can broadly be defined as open loop (where a fixed intensity is maintained until task disengagement), or closed loop (where a fixed distance is completed in the fastest time), which may involve whole-body or single-limb exercise. Closed loop exercise allows an analysis of how exercise intensity is self-regulated (i.e., pacing), and thus may better reflect the demands of competitive endurance performance. However, whilst this model can monitor changes in pacing, this is often at the expense of detecting subtle differences in the measured physiological or psychological variables of interest. Open loop exercise solves this issue, but is limited by its more restrictive exercise model. Nonetheless, much can be learnt from both experimental approaches when these constraints are recognized. Indeed, both models appear equally effective in examining changes in performance, and so the researcher should select the exercise model which can most appropriately test the study hypothesis. Given that a multitude of both internal (e.g., muscle fatigue, perception of effort, dietary intervention, pain etc.) and external (e.g., opponents, crowd presence, course topography, extrinsic reward etc.) factors likely contribute to exercise regulation and endurance performance, it may be that both models are required to gain a comprehensive understanding.

Consequently, this research topic seeks to bring together papers on endurance performance from a variety of paradigms and exercise models, with the overarching aim of comparing, examining and integrating their findings to better understand how exercise is regulated and how this may (or may not) limit performance. To explore new frontiers, we welcomed the submission of original research, review and perspective articles on endurance performance, which specifically consider the scope and impact of their findings in the broader context of exercise regulation.

## Topic content

This resulted in the acceptance of 24 papers (14 original research papers, 4 perspectives, 4 mini-reviews, a review, and an opinion) written by in total 84 contributing authors. Overall, the topic combines physiological with psychological viewpoints and papers explore closed-loop as well as open-loop exercise. Research papers from a predominantly physiological perspective were all directed toward a better understanding of endurance performance and its limitations, and/or directed toward optimizing endurance performance, and incorporated a wide range of methods.

## Fatigue and recovery

Fatigue and recovery were covered by several papers. VO_2_ kinetics and recovery in intermittent exercise was explored by Barbosa et al. They found that endurance performance was negatively influenced by active recovery only during shorter high-intensity intermittent exercise, though probably unrelated to differences in VO_2_ kinetics. Froyd et al. explored the critical fatigue threshold that has been proposed to limit endurance performance via inhibitory feedback from the group III and IV muscle afferents. They found that subjects did not terminate knee-extensor exercise at task failure because they had reached a critical threshold in peripheral fatigue and the existence of a critical peripheral fatigue threshold during intermittent isometric exercise to task failure with the knee extensors can thus be questioned. Also Neyroud et al. explored the critical fatigue threshold in their perspective article, highlighting the importance of considering interpretation of individual data and not only of group means. Muscle oxygenation, perceived fatigue and recovery were explored in speed skating by Hettinga et al. Patterns of reoxygenation and deoxygenation in the working muscles during a race are different for long-track and short-track speed skating, providing with more insights into the mechanistic physiological principles relevant for performance and recovery in elite athletes in different sports, and on how technical factors are impacting on those. Finally, Pageaux and Lepers explored mental and physical fatigue in their mini-review, and identified perception of effort as the variable altered by both prior physical exertion and mental exertion, that should be included in future studies.

## The role of the brain

Two experimental studies focused on the role of the brain in the regulation of exercise intensity. Hibbert et al. explored transcutaneous electrical nerve stimulation (TENS) effects on exercise-induced muscle pain, pacing strategy, and performance during a 5-km cycling time trial. Effects were found to be non-significant, and effectiveness of TENS could be questioned. There were indications that there was a possible effect at the start of the trials. Pires et al. explored cerebral regulation in different maximal aerobic exercise modes. Primary motor cortex activation was preserved throughout exercises, suggesting that central factors are at least partly centrally–coordinated. Angius et al. mini-reviewed the ergogenic effect of transcranial direct current stimulation on exercise performance, showing promising opportunities. However, also here it came forward that given the uncertain mechanisms and the inconsistency of outcomes of tDCS prior to exercise, the use of tDCS in exercise should be treated with some caution and future research is needed.

## Training physiology of endurance performance

Four experimental papers focused on training physiology of endurance performance. Schoenmakers et al. demonstrated that high intensity upper body interval training (HIIT) resulted in larger training effects compared to continuous training, and recommended to incorporate HIIT sessions in training regimes of recreationally active and trained handcyclists. De Araujo et al. discussed effects of HIIT they had found on hormones, metabolites, the anti-oxidant system, glycogen concentration and aerobic performance adaptations in rats into the training context of endurance runners. Guy et al. focused on effects of heat training on both endurance performance and biomarkers associated with inflammatory and immune system responses. Heat training enhanced performance and did not pose a substantial challenge to the immune system. Veldman et al. explored effects of neuromuscular electrical stimulation training on endurance performance, potentially particularly relevant for individuals with muscle weakness or patients who cannot perform voluntary contractions.

## Limits of human endurance performance: physiology and personality traits

Limits of human performance were addressed in a mini-review assessing the impact of age on physiological parameters, overviewing research on master athletes from Lepers and Stapley This paper strongly focused on physiological characteristics, where Schiphof-Godart et al. also included a psychological perspective to explore training behavior. They outline the possible influence of an athlete's passion in sport related to their exercise behavior and decision-making related to the regulation of exercise intensity. They conclude that taking into account athletes' passion could therefore be a useful tool for adequate coaching and monitoring of athlete well-being.

## The psychological perspective: deception studies and importance of the environment

From a psychological perspective, deception was a popular topic. Taylor and Smith demonstrated that mid-event pace deception can have a practically meaningful effect on multi-modal endurance performance, though the relative importance of different psychophysiological and emotional responses remains unclear. Williams et al. explored deceptive manipulation of competitive starting on several psychological and physiological parameters. Results demonstrated that with no detriment to performance time, but less physiological strain and more positive psychological perceptions, a pacing strategy adopting a slower start could be considered more beneficial during a stimulated 16.1 km cycling time trial. Jones et al. showed that time trial improvements were not sustained following acute provision of challenging and deceptive feedback. The presence of the pacer rather than the manipulation of performance beliefs acutely facilitated time trial performance and perceptual responses. This is in line with suggestions in the perspective of Hettinga et al., in which the science behind head-to-head competition was explored. They conclude that athlete–environment interactions are crucial factors in understanding the regulation of exercise intensity when racing against other competitors or pacers. Also Skorski et al. mention that environmental factors as important. In their mini-review, they conclude that pacing manipulations should be explored to further understand the complexity of how humans regulate pace.

When environmental factors are crucial, also the availability of feedback needs to be considered. Smits et al. examined the influence of the absence of commonly available task-related feedback on effort distribution and performance in experienced endurance athletes. They demonstrated that prior knowledge of task demands together with reliance on bodily and environmental information can be sufficient for experienced athletes to come to comparable time trial performances. In their meta-analysis, Davies et al. explored the effects of environmental feedback interventions on pacing. In line with the above studies, 26 cycling studies demonstrated environmental effects of hypoxia, thermal aspects and feedback on pacing and performance.

## The role of cognition in pacing

Also, cognitive aspects in pacing were covered. Van Biesen et al. explored in their original research study if the regulation of exercise intensity during competitive track races was different between runners with and without intellectual impairment. Runners with intellectual impairment have difficulties to efficiently self-regulate their exercise intensity. Their limited cognitive resources may constrain the successful integration of appropriate pacing strategies during competitive races, and establishes the role of cognitive factors in pacing and the regulation of exercise intensity. Brick et al. provided a cognitive perspective on self-regulation and endurance performance in their perspective article. They highlighted the roles of attentional focus, cognitive control, and metacognition in self-regulated endurance performance and mental fatigue. Mental fatigue was further explored in the study of Head et al. focusing on exploring cognitive fatigue in an experimental study. The authors found a decreased Time-on-Task in bodyweight resistance training exercise tasks.

## Conclusion

Recently, many researchers have focused on proposing frameworks to better understand the regulation of exercise intensity (Noakes, 1997; Marcora, 2008; Foster et al., 2009; Millet, 2011; Renfree et al., 2014; Smits et al., 2014; Hettinga et al.; Micklewright et al., 2017; St Clair Gibson et al., 2017; Venhorst et al., 2017). This research topic supports the notion that both internal and external variables need to be incorporated in frameworks exploring the regulation of exercise intensity. Both physiology and psychology are crucial for endurance performance, and aspects such as competitive environment, cognition and fatigue seem to be requisite to understand the regulation of exercise intensity. As yet, the way in which these factors interact in determining endurance performance is not fully understood.

The 24 papers comprising this research topic all explored the mechanisms involved in the regulation of exercise intensity. This issue was addressed within the context of a single bout of exercise, and across longer periods of time as is the case with long term changes in performance in masters athletes. As a whole, the papers have contributed to further understanding of fatigue & recovery, the role of the brain in regulatory processes, the relationship between physiological training responses and endurance performance, the limits of human performance and the influence of personality traits on endurance performance, and lastly the influence of environment, deception, and cognition on pacing. The number and range of issues considered within the broad subject of the regulation of exercise performance illustrates the complexity of the topic. Indeed, it is clear that (as stated in the introduction) a reductionist approach to understanding the regulatory process is unlikely to be sufficient. Although the papers comprising this research topic have greatly contributed to furthering our understanding of the key issues, it is still not clear how factors are “weighted” in terms of the extent to which they inform exercise regulation. Papers in this research topic alone have suggested that physiological status, psychological traits, and interactions with other competitors are all important. Researchers are encouraged to address the relative importance of these individual contributory factors in informing acute and chronic whole body behavior and performance during endurance exercise.

## Author contributions

FH drafted and finalized the manuscript. ARM, AR, BP, and HJ significantly contributed to the drafts toward the final product and critically reviewed the manuscript. All authors (FH, ARM, AR, BP, HJ, JC, and DM) provided valuable comments, thoughts and insights throughout the entire process of this topic that contributed to the final draft of this editorial, and approved the final version.

### Conflict of interest statement

The authors declare that the research was conducted in the absence of any commercial or financial relationships that could be construed as a potential conflict of interest.
